# Development of an Optically Induced Dielectrophoresis (ODEP) Microfluidic System for High-Performance Isolation and Purification of Bacteria

**DOI:** 10.3390/bios13110952

**Published:** 2023-10-25

**Authors:** Po-Yu Chu, Chia-Ming Yang, Kai-Lin Huang, Ai-Yun Wu, Chia-Hsun Hsieh, A-Ching Chao, Min-Hsien Wu

**Affiliations:** 1 Graduate Institute of Biomedical Engineering, Chang Gung University, Taoyuan City 33302, Taiwan; d000018394@cgu.edu.tw (P.-Y.C.); m1031009@cgu.edu.tw (K.-L.H.); m1031001@cgu.edu.tw (A.-Y.W.); 2 Department of Electronic Engineering, Chang Gung University, Taoyuan City 33302, Taiwan; cmyang@mail.cgu.edu.tw; 3 Institute of Electro-Optical Engineering, Chang Gung University, Taoyuan City 33302, Taiwan; 4 Biosensor Group, Biomedical Engineering Research Center, Chang Gung University, Taoyuan City 33302, Taiwan; 5 Department of Neurosurgery, Chang Gung Memorial Hospital at Linkou, Taoyuan City 33302, Taiwan; 6 Department of Materials Engineering, Ming Chi University of Technology, New Taipei City 243303, Taiwan; 7 Division of Hematology/Oncology, Department of Internal Medicine, Chang Gung Memorial Hospital at Linkou, Taoyuan City 33302, Taiwan; wisdom5000@cgmh.org.tw; 8 Division of Hematology/Oncology, Department of Internal Medicine, New Taipei Municipal TuCheng Hospital, New Taipei City 236017, Taiwan; 9 Department of Neurology, Kaohsiung Medical University Hospital, Kaohsiung City 80756, Taiwan; 10 Department of Neurology, College of Medicine, Kaohsiung Medical University, Kaohsiung City 80756, Taiwan

**Keywords:** optically induced dielectrophoresis (ODEP), microfluidic system, bacteria, isolation, purification

## Abstract

For the rapid detection of bacteria in a blood sample, nucleic acid amplification-based assays are believed to be promising. Nevertheless, the nucleic acids released from the dead blood cells or bacteria could affect the assay performance. This highlights the importance of the isolation of live bacteria from blood samples. To address this issue, this study proposes a two-step process. First, a blood sample was treated with the immuno-magnetic microbeads-based separation to remove the majority of blood cells. Second, an optically induced dielectrophoresis (ODEP) microfluidic system with an integrated dynamic circular light image array was utilized to further isolate and purify the live bacteria from the remaining blood cells based on their size difference. In this work, the ODEP microfluidic system was developed. Its performance for the isolation and purification of bacteria was evaluated. The results revealed that the method was able to harvest the live bacteria in a high purity (90.5~99.2%) manner. Overall, the proposed method was proven to be capable of isolating and purifying high-purity live bacteria without causing damage to the co-existing cells. This technical feature was found to be valuable for the subsequent nucleic-acid-based bacteria detection, in which the interferences caused by the nontarget nucleic acids could be eliminated.

## 1. Introduction

The isolation and purification of bacteria from clinical samples (e.g., blood) is fundamental for subsequent applications such as the detection of infectious diseases, identification of bacteria and selection of antibiotics [1,2,3,4]. Among the applications, the rapid detection of bacteria in a blood sample is important clinically. Sepsis, a fatal bloodstream infection (BSI) disease with a high mortality rate of 25%, can cause the infection of at least 49 million people worldwide [5]. For identifying the pathogenic bacteria causing sepsis and their antibiotic susceptibility (i.e., antimicrobial susceptibility test (AST)), a blood culture (BC) based method is conventionally utilized for bacteria detection in clinical settings [1,2,3,4,6,7]. Although blood cultures are easy to perform, they often require 40 to 80 mL of whole blood (approximately 2 to 4 blood cultures) to possibly detect pathogens from blood samples [1,3,8]. More importantly, BC is not only time-consuming (e.g., incubation time: 5~7 days [3,6,7,8]) but it is also difficult to detect the pathogenic bacteria with a slow-growing nature [4,7,9]. Furthermore, the BC-based method normally requires the associated techniques for the following bacteria purification (e.g., solid medium-based subculture, immunomagnetic microbead-based capture or microfluidic chip [3,4,6,10]) and for the final assays (e.g., PCR-, FISH-, MALDI-TOF MS-, Gram’s stain-based assays [2,3,4,7,10]) to identify pathogenic bacteria or to test the antibiotic susceptibility [2]. These technical hurdles make it difficult to provide septic patients with antibiotic treatment efficiently and precisely, particularly within 6 h of the onset of persistent hypotension induced by septic shock, which is reported to greatly improve the survival rate of patients [9,11].

For the rapid detection of sepsis, several emerging techniques (e.g., microfluidic-based systems, immunoaffinity-based methods and nucleic acid amplification tests (NAAT)) attempt to isolate or detect pathogenic bacteria directly from whole blood to save time spent on the conventional blood culture [4,6,7,9]. Due to the lack of blood culture for the expansion of the bacteria number, however, the above-mentioned techniques must first overcome the dilemma of only a small amount of bacteria in the blood sample of septic patients [6,7,9,12]). Among the emerging techniques, the detection of sepsis based on NAAT is believed to be promising, which can not only be used to identify pathogenic bacteria species but also to evaluate their antibiotic susceptibility [4,7,9]. For NAAT-based identification of pathogenic bacteria and AST, however, the nontarget nucleic acids released by a large number of blood cells and the PCR inhibitors (e.g., erythrocytes’ hemoglobin, white blood cells’ (WBCs) lactoferrin, immunoglobulin or enzymes [4,7,9,13]) existing in a whole blood sample could affect with the NAAT-based assays in terms of their detection accuracy as well as limitation of detection (LOD). Moreover, the target DNA released by the dead bacteria in a whole blood sample could also lead to false positive detection, which could in turn cause misleading results for clinical diagnosis (e.g., infection diagnosis) or clinical decision-making (e.g., the selection of antibiotics) [4,7,9]. As a whole, the facts abovementioned highlight the need for the isolation and purification of live bacteria from a complex whole blood sample without causing damage to the surrounding cells for the subsequent reliable and accurate bacteria detection or AST via NAAT.

Thanks to the recent progress and advantages of microfluidic technology in various fields (e.g., self-assembly and biosensing [14,15,16,17]), the microfluidic systems integrating different working mechanisms (e.g., acoustophoresis [18], inertial focusing [19], immunomagnetic separation [20] or dielectrophoresis (DEP) [21]) have been developed for the isolation and purification of pathogenic bacteria from BSI blood samples without causing damage to the surrounding WBCs. This technical feature could therefore alleviate the interference of nontargeted nucleic acids in subsequent NAAT detection [4,6,21]. Among them, the DEP-based microfluidic system has been successfully demonstrated to separate the target bacteria from saponin-treated whole blood samples. Its results also showed that the isolated and purified bacteria kept a high degree of bacterial viability [21]. Although the DEP force-based method is feasible for the isolation and purification of bacteria from a blood sample, this technique normally requires a technically demanding and costly process to fabricate a specific metal microelectrode array that is for a particular application [22,23]. This requirement could restrict its practical applications.

To address the technical issue, microparticle manipulation based on optically induced dielectrophoresis (ODEP) is believed to be a promising alternative technique for the task. Basically, the working principle of ODEP-based microparticle manipulation is similar to that of DEP-based one, apart from the utilization of optical images as virtual electrodes to replace the metal microelectrodes in the DEP-based technique [22,23,24,25]. ODEP-based microparticle manipulation was first presented in 2005 [22] and well described previously [23,24,25]. Briefly, an electric field is exerted in the thin solution layer of an ODEP system to electrically polarize the microparticles within the solution. After that, light illuminates the photoconductive substrate of an ODEP system causing a decrease in electrical impedance in the specific light-illuminated zone and therefore leading to a local nonuniform electric field. In an ODEP system, the interaction between the electrically polarized microparticles and the nonuniform electric field generated via specific light illumination can generate the ODEP force. For practical microparticle manipulation, therefore, scientists can simply use a moving light image to dynamically manipulate microparticles. This technical feature could contribute to a more flexible microparticle manipulation compared to the conventional DEP technique, which requires prefabricated microelectrodes [22,23,24,25]. The technique of ODEP-based microparticle manipulation has been successfully presented for a wide variety of biological applications, mainly in the field of sorting, separation or purification of cells (e.g., the separation of dead and living cells [22], the isolation of rare cells in blood (e.g., circulating tumor cells (CTCs) [24]) and the sorting and separation of bacteria [23] with different drug resistance). Additionally, the ODEP-based manipulation of cells under an appropriate electric field condition has been proven not to affect the property and viability of biological cells [25]. All these facts demonstrate that the technique of ODEP-based cell manipulation is suitable for the isolation and purification of bacteria from a biological sample.

In order to isolate and purify the live bacteria from the blood samples of septic patients, this study proposes a two-step process. First, the pre-enrichment of whole blood samples using well-known centrifugation [26], specific filtering membrane [26] or the immuno-magnetic microbeads-based separation technique [24] was designed to remove the majority of unwanted blood cells and reduce the working volume. In the second step, an ODEP microfluidic system was designed to further isolate and purify the live bacteria from the remaining blood cells in a continuous and high-performance manner. In the proposed ODEP microfluidic system, a dynamic circular light image array consisting of multicolumn circular light images was designed in the main microchannel of a microfluidic system to remove the unwanted blood cells remaining in the treated blood sample in a continuous manner. Through this operation, the live bacteria can be effectively separated and collected in a high-purity manner. The key working mechanism is based on the fact that the ODEP force generated on microparticles is proportional to the cube of their radius [24,25]. Therefore, the ODEP force generated on the bacteria (e.g., the diameter of *E. coli:* around 2 μm [23]) and WBCs (diameter: 9~18 μm [27]), the remaining cells in the treated blood sample, would be different. As a result, the designed ODEP-based dynamic circular light image array was capable of sorting and separating the WBCs and bacteria in an effective manner via ODEP-based cell manipulation.

In this study, the SW620 cancer cells, estimated to have a similar size and thus ODEP force as that of WBCs [24], were used as a stable test model instead of using human WBCs. The optimum ODEP condition for the effective sorting and separation of live SW620 cancer cells and live bacteria (the use of *E. coli* as a test model) without affecting cell and bacteria viability was first determined. This was followed by a series of experimental works to determine the optimum operating conditions (i.e., diameter of circular light image, the gap between circular light images, the optimum combination of sample flow rate and the moving velocity of circular light images as well as the optimum design of dynamic circular light image array) for the high-performance isolation and purification of bacteria. In the subsequent performance evaluations, the SW620 cancer cell suspension spiking with *E. coli* was prepared to mimic the blood sample of septic patients treated with the first step process as aforementioned, in which the RBCs and 99.9% of WBCs were removed. Based on the test model, the performance of the proposed ODEP microfluidic system for the isolation and purification of bacteria was evaluated. The results revealed that the proposed method was able to harvest the live bacteria with purity as high as 90.5~99.2% within the experimental conditions explored. As a whole, the presented method was proven to be capable of isolating and purifying high-purity live bacteria without causing damage to the co-existing cells. This technical advantage is found to be particularly meaningful for the subsequent NAAT-based bacteria detection or AST, in which the interferences caused by the nucleic acids released from the dead cells or dead bacteria could be eliminated.

## 2. Materials and Methods

### 2.1. The ODEP Microfluidic System

**Figure 1:**
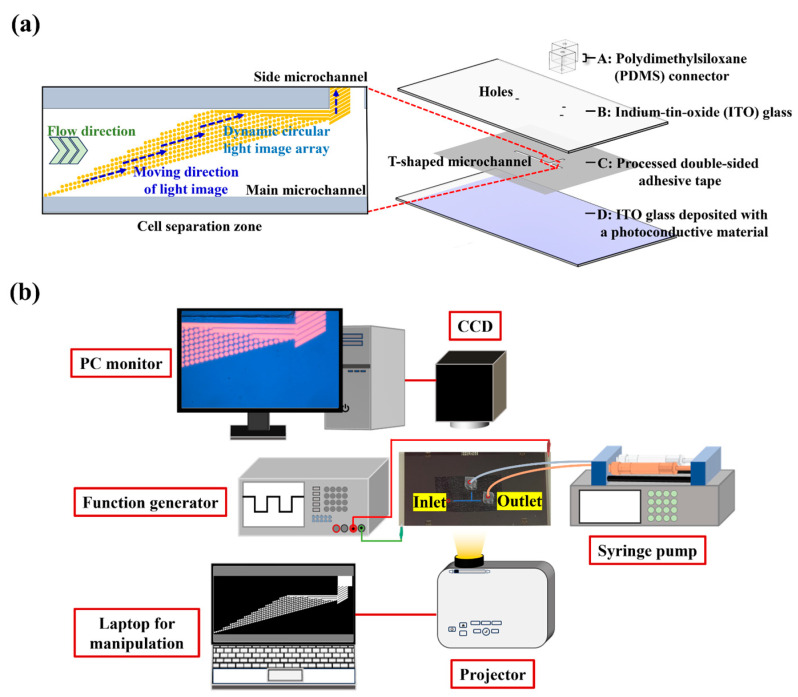
Schematic illustration of (**a**) the laminated structure of the ODEP microfluidic system and the close-up view of cell separation zone and (**b**) the overall experimental setup.

In this study, a microfluidic system with an integrated ODEP mechanism was utilized for the size-based isolation and purification of bacteria from a processed cell suspension. Its structure is shown in Figure 1a. The microfluidic system primarily consists of a T-shaped microchannel as indicated in Figure 1a. Its main microchannels (length (L): 20 mm, width (W): 1 mm, height (H): 50 µm) were designed for the transportation of a prepared sample and the collection of bacteria. The side microchannel (L: 10 mm, W: 400 µm, H: 50 µm) was used for the collection of the separated cells. In this work, three holes (diameter (D): 1.0 mm, H: 0.70 mm) on an ITO glass layer as indicated were designed to connect the T-shaped microchannel to the outside world via tubes. Moreover, a dynamic circular light image array was designed in the defined cell separation zone (L: 4.2 mm, W: 1 mm) of the main microchannel (Figure 1a) for the continuous separation of the cells and bacteria. The separated cells and bacteria were then collected through the side microchannel and downstream part of the main microchannel, respectively. Structurally, the microfluidic system is composed of four layers (layer A: a custom-made polydimethylsiloxane (PDMS) connector; layer B: an up-side-down indium-tin-oxide (ITO) glass; layer C: a processed double-sided adhesive tape (H: 50 µm); layer D: an ITO glass deposited with a photoconductive material (i.e., a 20-nm-thick n^+^ hydrogenated amorphous silicon layer (n^+^ a-Si:H) and a 1 μm-thick intrinsic hydrogenated amorphous silicon (a-Si:H) layer)) as indicated in Figure 1a.

In this work, the approaches for the fabrication and assembly of the microfluidic system were the same as those described previously [24]. Moreover, the schematic illustration of the entire experimental setup for the operation of the ODEP microfluidic system is shown in Figure 1b, which was also well described previously [24]. In short, the sample flow in the main microchannel was driven by a syringe pump (KDS LEGATO 180, KD Scientific, Holliston, MA, USA). To achieve the ODEP-mechanism-based cell and bacteria manipulation for separation and purification purposes, a function generator (AFG-2125, Good Will Instrument Co., Ltd., New Taipei City, Taiwan) was used to create an alternating current (AC) voltage between the two ITO electrodes (Figure 1a). In this work, a computer-controlled projector (EB-X05, Epson, Suwa, Japan) was used to illuminate specific light images (e.g., dynamic circular light image array) onto the bottom a-Si:H layer of the ODEP microfluidic system for the cell and bacteria manipulation. The observation of the cell and bacteria manipulation process was achieved via a CCD-equipped microscope (Zoom 160, OPTEM, Medina, OH, USA).

### 2.2. The Mechanism of the Proposed ODEP-Based Dynamic Circular Light Image Array for the Continuous Separation of Cells and Bacteria

The ODEP mechanism for cell manipulation has been described earlier in the introduction section [22,23,24,25]. The ODEP force acting on a microparticle can be expressed by Equation (1) below (r, ε_0_, ε_m_, ∇|E|^2^, and Re[f_CM_]: the microparticle radius, vacuum permittivity, relative permittivity of working solution, gradient of the exerted electrical voltage squared and real part of the Clausius–Mossotti factor (f_CM_), respectively) [24,25]:

F_DEP_ = 2πr^3^ε_0_ε_m_Re[f_CM_]∇|E|^2^ (1)

**Figure 2:**
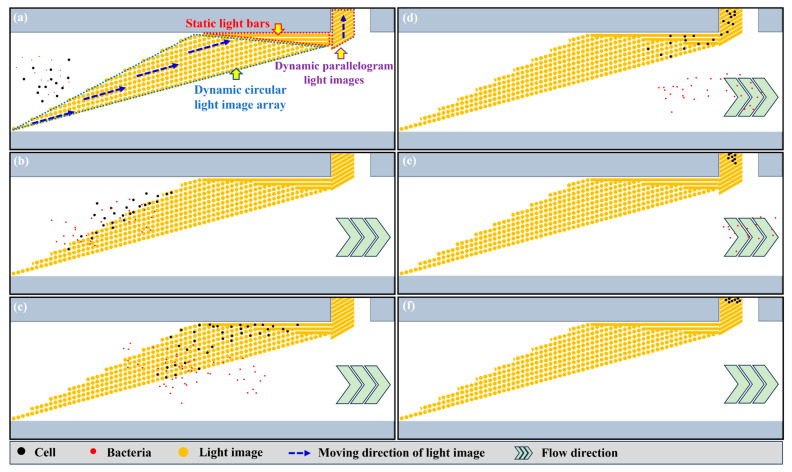
Schematic illustration of the overall processes for the isolation and purification of bacteria. (**a**) The dynamic circular light image array functioning both as a virtual filter (i.e., sorting and separation function) and as a virtual conveyor (transportation function) was designed. In addition, three static parallel light bars and dynamic parallelogram light images were designed to further transport the cells to the side microchannel. (**b**–**f**) Cells (the black dots) were trapped within the circular light images individually and were transported by the dynamic circular light images, three static parallel light bars and the designed dynamic parallelogram light images to the side microchannel. Conversely, the bacteria (the red dots) were not trapped by the circular light image array and flowed through the circular light image array directly. They were then collected at the downstream part of main microchannel.

It can be observed from Equation (1) that the ODEP force acting on a manipulated microparticle is proportional to its cubic radius. According to this fact, ODEP-based cell manipulation could be used to separate the unwanted WBC (diameter: 9~18 μm [27]) and the bacteria (e.g., diameter of *E. coli:* around 2 μm [23]) of interest in the treated blood sample based on their significant size differences. For achieving a high-efficiency operation, moreover, a specific dynamic light image array consisting of circular light images was designed in the defined cell separation zone (Figure 1a) for the continuous separation of cells and bacteria. Figure 2 schematically illustrates the overall operation process. In the design, the dynamic circular light image array not only served as a virtual filter for sorting and separating cells and bacteria based on their size difference but also worked as a virtual and multilevel conveyor that continuously transported the unwanted cells to the side microchannel as shown in Figure 2a. When the cells reached the dynamic circular light image array (Figure 2b), briefly, they were trapped within the circular light images individually due to the action of ODEP force. The trapped cells were then transported by the dynamic circular light images to one side of the main microchannel where three static and parallel light bars (W: 45 µm; L: 1526, 889, 464 μm for the three static light bars, respectively) (Figure 2a) were designed for further collecting and guiding the transported cells to the entrance of side microchannel (Figure 2b–d). After that, the cells reaching the entrance of the side microchannel were further transported to the side microchannel via the designed dynamic parallelogram light images (W: 45 μm; L: 468 μm; angle: 30°; columns: 10) as shown in Figure 2d–f. Differently from the cells, the bacteria were not trapped by the designed circular light image array mainly due to their smaller size compared to the cells. Therefore, the bacteria could flow through the circular light image array directly and then be collected at the downstream part of the main microchannel as shown in Figure 2b–f. Based on the design, overall, the bacteria can be effectively separated and isolated from a treated blood sample containing cells.

### 2.3. The Optimization of Operation for the Separation and Purification of Bacteria

Instead of using human WBCs, the SW620 cancer cell line (diameter: 15.6 ± 1.4 μm), microscopically estimated to have a similar size to WBCs (diameter: 9~18 μm [27]), was used as the model cells representing the remaining cells in the treated blood sample for establishing a stable test model purpose. In this study, the size (i.e., diameter) of the SW620 cancer cells and *E. coli* (as a test bacteria model) was first measured microscopically to ensure they had significant differences. For achieving ODEP operation, moreover, the basic ODEP operating conditions (i.e., the electric voltage: 10 peak-to-peak voltage (Vpp) and the ODEP working solution: 0.05% (*w*/*v*) BSA in 9.5% (*w*/*v*) sucrose solution (conductivity: 9.5~11.0 μS cm^−1^)) were adopted. In order to determine the optimum frequency of the AC electric voltage for the effective separation performance, the ODEP force acting on the SW620 cancer cells and *E. coli* was assessed under various frequency conditions (e.g., 1, 2, 3, 4, and 5 MHz). For this evaluation, the ODEP manipulation force, the net force between the ODEP force acting on the manipulated cell and the friction force acting on such a moving cell, was experimentally assessed [24,25]. In this study, the evaluation of ODEP manipulation force was based on the measurement of the maximum velocity of a moving light image (e.g., the circular light image with a diameter of 45 μm) that can manipulate a cell. To ensure that the viability of the isolated bacteria was not affected by the ODEP operation, furthermore, the bacterial viability was assessed before and after ODEP operation (i.e., the magnitude and frequency of electric voltage: 10 Vpp and 3 MHz, respectively) using a Live/Dead BacLight Bacterial viability kit [28].

Apart from the fundamental conditions abovementioned, the other operating conditions relevant to the design of the dynamic circular light image array (i.e., the diameter of circular light images, the gap between circular light images and the optimum combination of sample flow rate and the moving velocity of circular light images) were determined based on experimental tests. In order to find out the optimum size of the circular light images for manipulating the SW620 cancer cells, briefly, the maximum velocity of the dynamic circular light images with varied diameters (e.g., 30, 45, 60 and 75 μm) that can manipulate the cancer cells was evaluated experimentally. For the effective separation of the SW620 cancer cells from a sample flow, moreover, the optimum gap between the circular light images was determined. In this work, the cell trapping rates of the cancer cell suspension (5 × 10^4^ cells mL^−1^) flowing through a single column of dynamic circular light images (i.e., diameter of light image: 45 μm; gap between light images: 5, 10, 15, 20, 25 and 30 μm; moving velocity of circular light images: 200 μm s^−1^) slantingly lying across the main microchannel (angle to the sample flow: 15°) were assessed experimentally. For determining the optimum combination of the sample flow rate and the moving velocity of circular light images, furthermore, the evaluation of cell trapping rates as abovementioned was carried out under the sample flow rate range and the moving velocity range of circular light images of 0.5~2.5 μL min^−1^ and 50~400 μm s^−1^, respectively.

### 2.4. Performance Evaluation of the Proposed ODEP-Based Dynamic Circular Light Image Array for the Continuous Isolation and Purification Bacteria

After the aforementioned operation conditions were determined, the performance of dynamic circular light image arrays with two different designs (i.e., the arrays with the uniform front line or jagged front line designs (i.e., the design shown in Figure 2)) was compared in terms of their ability to separate and isolate the unwanted cells from a sample flow. The purpose was to select one design of circular light image array from the two designs as aforementioned. For this performance evaluation, the cell suspension sample (cell concentration: 2.5 × 10^4^~2.0 × 10^5^ cells mL^−1^) of SW620 cancer cells was prepared. The prepared sample was then loaded into the proposed ODEP microfluidic system and followed by the cell separation and isolation operation as illustrated in Figure 2. In this work, the cell recovery (i.e., (the cell numbers obtained in the side microchannel/the total cell numbers originally loaded into the ODEP microfluidic system) × 100%) was then measured. 

After the design of the dynamic circular light image array was determined, its performance for the isolation and purification of bacteria from a mixture sample containing bacteria (i.e., *E. coli*) and cells (i.e., SW620 cancer cells) was experimentally evaluated. In this study, the mixture sample containing bacteria and cells that mimic the treated blood sample of septic patients was first prepared by adding varied ratios of bacteria into the cells prestained with calcein red-orange fluorescent dye (CellTraceTM Calcein Red-Orange, C34851, Invitrogen, Carlsbad, CA, USA). The prepared sample was then treated with operation as illustrated in Figure 2. After that, the sample collected via the downstream part of the main microchannel was assayed in terms of the purity of the bacteria harvested. For this evaluation, half of the collected sample was assayed via fluorescent microscopic observation to quantify the cell number of the SW620 cancer cells prestained with fluorescent dye. In this work, moreover, another half of the collected sample was used for a 12~24 h bacteria culture so as to quantify the bacteria number (i.e., counting of bacterial colony forming units, CFU) obtained in the harvested sample. After the quantification of the cells and bacteria in the harvested sample, the purity of the bacteria obtained was then calculated (i.e., the bacteria purity = (the number of bacteria/the total number of bacteria and cells) × 100%).

### 2.5. Statistical Analysis

In this study, the results were presented as the mean ± standard deviation based on at least three experiments. One-way ANOVA was used to evaluate the effect of the operating condition explored on the outcomes. Tukey’s honestly significant difference (HSD) post hoc test was used to compare the differences between the two conditions explored when the null hypothesis of the ANOVA was rejected.

## 3. Results and Discussion

### 3.1. ODEP Operation Condition for the Separation of Bacteria and Cells without Causing Their Damage

**Figure 3:**
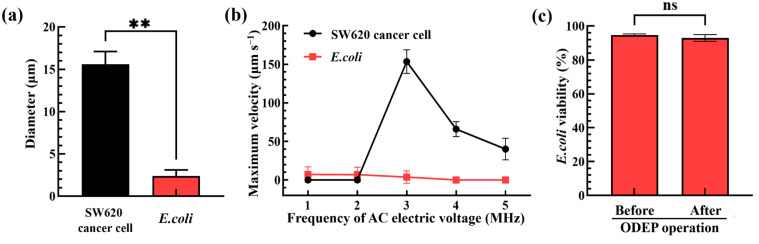
(**a**) Comparison of the size of SW620 cancer cells and *E. coli*, (**b**) the measured maximum velocity of circular light image that was able to manipulate the SW620 cancer cells and *E. coli* under varied frequencies (i.e., 1, 2, 3, 4 and 5 MHz) of AC electric voltage applied, and (**c**) the evaluation of bacterial viability before and after ODEP operation. (** Significant difference (*p* < 0.01); ns: Not significant).

To realize the working mechanism as described in Figure 2, the appropriate operation conditions of ODEP were determined. First, the optimal ODEP manipulation conditions for the separation of bacteria and cells without causing damage were explored. As shown in Figure 3a, the diameters of SW620 cancer cells (i.e., the test model cells representing the WBCs in a real blood sample) and *E. coli* (i.e., the test model bacteria representing the pathogenic bacteria in a real blood sample) were first microscopically measured to be 15.7 ± 1.5 μm and 2.4 ± 0.7 μm, respectively, which were evaluated to have a statistical difference (*p* < 0.01). The significant size difference ensured that the cells and bacteria could be effectively sorted and separated based on the ODEP-based microparticle manipulation as described previously [24,25]. In order to determine the appropriate frequency of AC bias (applied voltage: 10 Vpp) for effective separation performance, moreover, the ODEP manipulation force of cells and bacteria was evaluated under different frequency conditions (e.g., 1, 2, 3, 4 and 5 MHz). For this evaluation, the maximum velocity of the circular light image that can manipulate the bacteria and cells, as an indicator of ODEP manipulation force [24,25], was experimentally measured. The results (Figure 3b) revealed that the phenomena of surface adhesion, aggregation or damage of cells and bacteria were observed (images not shown) when the frequency of the AC electric voltage applied was set at 1 and 2 MHz within the experimental conditions explored. The findings revealed that the frequency condition of 2 MHz or lower was not suitable for the task of ODEP-based cell and bacteria manipulation for this separation purpose. On the contrary, the abovementioned phenomena were not observed when the applied frequency was higher than 2 MHz. Within the frequency conditions tested (i.e., 3, 4 and 5 MHz), the maximum velocity of the circular light image that was able to manipulate the bacteria and cells decreased with the increase in frequency. Additionally, the difference between the maximum velocity of the circular light image that was able to manipulate the bacteria and cells was significant. This could be mainly due to the significant size difference between the bacteria and cells as shown in Figure 3a as the ODEP manipulation force and thus the maximum velocity of the circular light image that can manipulate cells or bacteria is proportional to their radius cubic as mentioned in Equation (1). Within the experimental conditions explored, the AC bias voltage with the magnitude and frequency of 10 Vpp and 3 MHz, respectively, was adopted, under which the difference in the maximum velocity of the circular light image that was able to manipulate the bacteria and cells was the most significant (i.e., the measured maximum velocities of light images that can manipulate the SW620 cancer cells and *E. coli*: 153.5 ± 15.4 and 3.8 ± 8.1 μm s^−1^, respectively). Under the abovementioned ODEP condition, furthermore, the viability of the biological cells was reported not to be affected by ODEP [25]. This fact is important, otherwise, the nucleic acids released by the dead or damaged cells caused by ODEP conditions could contaminate the bacteria sample harvested in the downstream part of the main microchannel. This contamination could in turn cause problems (e.g., the reduction of detection specificity or sensitivity) in its subsequent NAAT-based detection work. However, the impact of this selected ODEP operation condition (i.e., the AC bias voltage with the magnitude and frequency of 10 Vpp and 3 MHz, respectively) used in a previous study [25] on bacterial viability has not yet been investigated. To address this issue, the viability of *E. coli* before and after the ODEP operation under the abovementioned condition was evaluated. The results (Figure 3c) exhibited that the bacterial viability before and after ODEP operation showed no significant difference (*p* > 0.05). The bacterial viability remained as high as 91.3~95.4%. This finding again ensures that the bacteria sample harvested by the presented method would not be contaminated by the nucleic acids released by the dead or damaged bacteria caused by ODEP operation.

### 3.2. The Operation Condition of Dynamic Circular Light Image Array for Size-Based Separation of Cells and Bacteria

**Figure 4:**
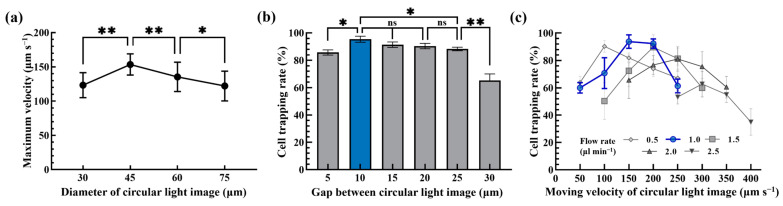
(**a**) The size (30, 45, 60 and 75 μm) effect of a circular light image on the maximum velocity of a circular light image that can manipulate a cell, (**b**) the effect of the gap (5, 10, 15, 20, 25 and 30 μm) between circular light images on the cell trapping rate of the cancer cell suspension flowing through a single column of dynamic circular light images (moving velocity of circular light images: 200 μm s^−1^; sample flow rate: 1.5 μL min^−1^) and (**c**) the combined effect of cell suspension flow rate (flow rate range: 0.5~2.5 μL min^−1^) and the moving velocity of circular light images (50~400 μm s^−1^) on the cell trapping rate of the cancer cell suspension flowing through a single column of dynamic circular light images (* significant difference (*p* < 0.05), ** significant difference (*p* < 0.01), ns: not significant).

After the above ODEP operation condition was determined, the operating conditions of the proposed dynamic circular light image array were then explored. In this work, briefly, the moving circular light images were mainly utilized to capture and transport the tested cells to one side of the microchannel as illustrated in Figure 2. Ideally, each circular light image was designed to capture and transport a single cell avoiding the aggregation of them and the resulting problems. To determine the optimal size of each circular light image in the dynamic circular light image array, the maximum velocities of circular light images with different diameters (i.e., 30, 45, 60 and 75 μm) that can manipulate the tested cells (i.e., SW620 cancer cells) were experimentally assessed. The results (Figure 4a) exhibited that the circular light images with a diameter of 45 μm had the highest ODEP maximum velocity (153.5 ± 15.4 μm s^−1^) and thus ODEP manipulation force compared to other conditions tested. This finding could be explained by the fact that the size of the light image used could play a role in the ODEP force generated on a manipulated microparticle. Based on the evaluation, therefore, the circular light image with a diameter of 45 μm was designed in the following works.

In this study, moreover, multiple columns of dynamic circular light images slantingly lying across the main microchannel were designed to create the dynamic circular light image array as illustrated in Figure 2a. For each single column consisting of separate circular light images, it was designed to slantingly lie across the main microchannel with an angle of 15° to the sample flow. The design of the angle to the sample flow was based on the previous work [24] for maximizing the capture rate of cells when they flowed through the column of circular light images. Differently from the previous work [24], in which a static rectangular light image bar slantingly lying across the main microchannel was designed to capture the cells flowing through, this study utilized multiple columns of separate circular light images (i.e., the dynamic circular light image array) to enhance cell capture performance. In order to determine the optimum gap between 8014 he circular light images, the cell trapping rate of the cancer cell suspension (5 × 10^4^ cells mL^−1^) flowing through a single column of dynamic circular light images (e.g., gap between light images: 5, 10, 15, 20, 25 and 30 μm; moving velocity of circular light images: 200 μm s^−1^; sample flow rate: 1.5 μL min^−1^) slantingly lying across the main microchannel were assessed experimentally. The results (Figure 4b) showed that the cell trapping rate significantly (*p* < 0.01) decreased when the gap between the circular light images was higher than 25 μm. This phenomenon could be simply explained by the fact that a larger gap (e.g., 30 μm) between the light images could allow more cells (D: 15.7 ± 1.5 μm; Figure 3a) to pass through, resulting in a lower cell trapping rate. When the gap was as small as 5 μm, similarly, the cell trapping rate significantly (*p* < 0.01) declined compared to a gap of 10 μm. This phenomenon was mainly due to the fact that the circular light images were too close to effectively keep cells in the circular light images when they passed through, resulting in cell aggregation. This phenomenon significantly affected the cell trapping rate as shown in Figure 4b. Conversely, the cell trapping rate kept as high from 95.4 ± 2.2% to 90.4 ± 2.0% when the gap was in the range from 10 to 20 μm, which showed no statistical difference (*p* > 0.05). In this work, the 10 μm gap between the circular light images was selected to maximize the number of circular light images under the same column length. This could in turn increase cell trapping performance.

After determining the design of a single column of circular light images as mentioned above, the combined effect of the cell suspension flow rate (cell concentration: 10^4^ cells mL^−1^; flow rate range: 0.5~2.5 μL min^−1^) and the moving velocity of the circular light images (50~400 μm s^−1^) on the cell trapping rate was experimentally explored. The results (Figure 4c) revealed that the maximum cell trapping rate was only 62.8 ± 9.1% and 81.1 ± 8.9% when the sample flow rate was 2.0 and 2.5 μL min^−1^, respectively. This phenomenon could be due to the fact the flow velocity of the cells under the high flow rate range of 2.0~2.5 μL min^−1^ was much higher than that of 153.5 ± 15.4 μm s^−1^, which was the maximum velocity of a circular light image (D: 45 μm) that can manipulate the cells as shown in Figure 4a. In this situation, the designed circular light images might not be able to effectively attract and capture the cells flowing through. Within the flow rate conditions explored of 0.5~1.5 μL min^−1^, conversely, the cell trapping rate researched the high level of 90.3 ± 4.3%, 93.8 ± 4.9% and 92.3 ± 3.4% (e.g., the flow rate: 0.5 μL min^−1^ and moving velocity of circular light images: 100 μm s^−1^, or the flow rate: 1.0 μL min^−1^ and moving velocity of circular light images: 150 and 200 μm s^−1^, respectively). Considering the overall performances (e.g., the cell trapping rate, the stability of operation and the working throughput), the flow rate of cell suspension and the moving velocity of the circular light images were set at 1.0 μL min^−1^ and 200 μm s^−1^, respectively, for the following work, which was able to achieve the average cell trapping rate of 92.3 ± 3.4% based on triplicate experiments (Figure 4c).

### 3.3. Design of Dynamic Circular Light Image Array for High-Performance Separation of Cells

**Figure 5:**
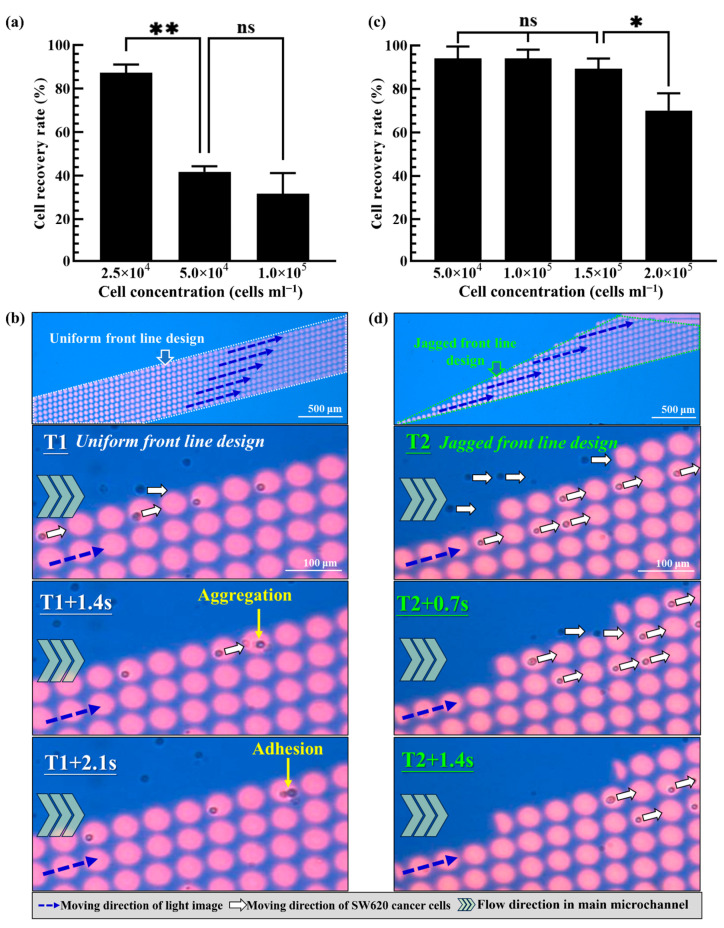
(**a**) The evaluation of cell recovery rate (i.e., the cell separation performance) of the proposed dynamic circular light image array with uniform front-line design under different cell concentration conditions as indicated, (**b**) continuous microscopic observations of the flowing cells trapped and transported by the designed dynamic circular light image array with uniform front-line design (cell aggregation and adhesion were observed), (**c**) the evaluation of cell recovery rate of the proposed dynamic circular light image array with jagged front-line design under different cell concentration conditions as indicated and (**d**) continuous microscopic observations of the flowing cells trapped and transported by the designed dynamic circular light image array with jagged front-line design (cell aggregation and adhesion were not observed). (* Significant difference (*p* < 0.05), ** significant difference (*p* < 0.01), ns: not significant).

Based on the fundamental evaluations mentioned above, the 45 μm diameter of the circular light images, 10 μm gap between the circular light images, the 15° angle of the column of circular light images to the sample flow, the 200 μm s^−1^ moving velocity of the circular light images and the 1.0 μL min^−1^ of the sample flow rate were determined. In the following work, the design of a dynamic circular light image array was explored. First, 10 columns of circular light images were parallelized to form a dynamic circular light image array with a uniform front line. The cell separation performance of such a design was first experimentally evaluated. In this work, an SW620 cancer cell suspension with different concentrations (i.e., 2.5 × 10^4^, 5.0 × 10^4^ and 1.0 × 10^5^ cells mL^−1^) was prepared and processed using the design of a dynamic circular light image array as mentioned above. Its cell separation performance was then evaluated in terms of the recovery of the cells in the side microchannel. Within the experimental conditions tested, the results (Figure 5a) revealed that the cell recovery rate decreased with an increase in the cell concentration. In this work, the cell recovery rate researched at the highest level of 87.3 ± 3.8% under the lower cell concentration conditions (i.e., 2.5 × 10^4^ cells mL^−1^). When the cell concentration was increased (i.e., 5.0 × 10^4^, and 1.0 × 10^5^ cells mL^−1^), the cell recovery rate significantly declined to the level of 41.8 ± 2.5% and 31.7 ± 9.5%, respectively, which showed no significant difference between them. This phenomenon was mainly due to the occurrence of cell aggregation resulting from the collision of cells at the first line of a dynamic circular light image array with a uniform front-line design. This phenomenon was observed in the photograph shown in Figure 5b and the Supplementary Video Clip (Supplementary Video S1). Overall, the phenomenon of cell aggregation and further adhesion could in turn affect the capability of the circular light images to capture and transport cells, and thus the resulting low cell recovery rate (i.e., the cell separation performance).

To tackle the technical hurdle, the dynamic circular light image array with a jagged front line as illustrated in Figure 2 was designed. A similar performance evaluation as mentioned above was carried out to assess its cell separation performance. The results (Figure 5c) demonstrated that the cell recovery rate remained high from 94.0 ± 5.5% to 89.3 ± 4.7% under the cell suspension concentration range of 5 × 10^4^~1.5 × 10^5^ cells mL^−1^. However, the cell recovery rate (i.e., 70.1 ± 7.9%) had a significant decrease when the cell concentration of the sample researched was 2.0 × 10^5^ cells mL^−1^. As a whole, the dynamic circular light image array with a jagged front line (i.e., Figure 2) was proven to have a higher cell recovery rate (i.e., the cell separation performance) and to have the capability to process the cell suspension sample with a higher cell concentration compared to that based on the previous design (Figure 5a,b). This outcome could be due to the fact that the latter design could allow the cells in the sample flow to be captured and transported independently as shown in Figure 5d and the Supplementary Video Clip (Supplementary Video S2). This design could therefore avoid the undesirable cell aggregation phenomenon as seen in Figure 5b and could improve the cell recovery rate (and thus the cell separation performance).

### 3.4. Performance of the Proposed ODEP Microfluidic System for the Continuous Isolation and Purification of Bacteria

**Figure 6:**
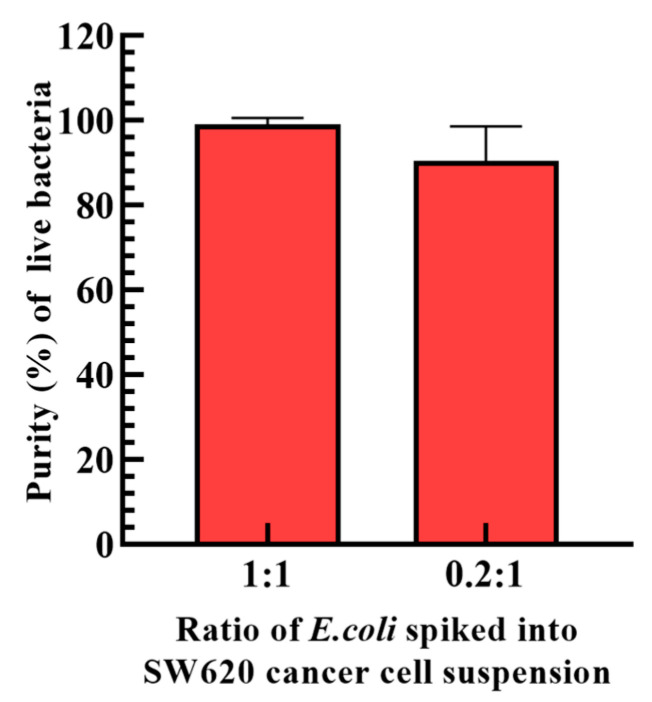
The evaluation of the purity of bacteria harvested after the proposed ODEP-based bacteria isolation and purification process (the ratio of *E. coli* in SW620 cancer cells: 1:1 and 0.2:1).

After the determination of the operation conditions (Figure 3 and Figure 4) and the design of the dynamic circular light image array (Figure 2 and Figure 5), the performance of the proposed ODEP microfluidic system for the isolation and purification of bacteria was experimentally assessed. In this work, *E. coli* was spiked into a cell suspension (1.5 × 10^5^ cells mL^−1^) of SW620 cancer cells prestained with calcein red-orange dye in a ratio of 1:1 and 0.2:1, which mimics a blood sample of septic patients treated with the first step operation (e.g., immuno-magnetic beads-based RBC and WBC separation) to remove the 99.9% of blood cells. The prepared sample was then loaded into the presented ODEP microfluidic system and followed by the operation as illustrated in Figure 2. Similarly to the illustration in Figure 2, the Supplementary Video Clip (Supplementary Video S3) showed that most of the SW620 cancer cells were effectively captured and transported to the side microchannel via the designed ODEP mechanisms including the dynamic circular light image array, the static light bars and the dynamic parallelogram light images (Figure 2). In addition, the phenomena of cell death, cell lysis and cell aggregation were not observed in Supplementary Video S3, which is in line with previous evaluations (i.e., Ref. [25] and Figure 5d, respectively). This also indicates that the method proposed in this study could largely reduce the cell death caused by the fluid shear stress [29] compared to the other microfluidic-based bacteria/cell isolation and purification schemes. The death of cells could lead to the release of other nontarget DNA or PCR inhibitors that might affect the performance of the following NAAT. After the ODEP-based isolation and purification process, moreover, the sample collected via the downstream part of the main microchannel was assayed in terms of the purity of the bacteria harvested. The results (Figure 6) revealed that the proposed method was able to harvest the live bacteria (Supplementary Figure S1 demonstrated the bacteria culture of the processed sample as well as the microscopic observation of cells in the original sample and the processed sample in the 1:1 case) with a purity as high as 90.5~99.2% within the experimental conditions explored. In previous studies, the purity of the bacteria isolation based on the DEP-[21] or acoustophoresis-[30] based microfluidic systems were reported to be 79 ± 3% and 95.65%, respectively. Compared to the previous techniques as mentioned above, this proposed method could harvest a high purity of the viable bacteria, facilitating the following NAAT. Meanwhile, the design, fabrication and operation of the proposed ODEP microfluidic systems are simpler, contributing to its practical applications. Overall, the presented method was proven to be capable of isolating and purifying high-purity live bacteria without causing damage to the co-existing cells. The technical advantage of the proposed protocol was found to be particularly meaningful for the subsequent NAAT-based bacteria detection or AST, in which the interferences caused by the nucleic acids released from the dead cells or dead bacteria could be eliminated. Although the obtained bacteria are sufficient for the subsequent NAAT in this proof-of-concept study (e.g., the limitation of detection of the commonly-used NAAT technique like RPA, QPCR is around 1–10 CFU bacteria [31,32]), the improvement in the operation throughput is our important future work. In addition, considering the complicated situation of real blood samples, the use of the proposed protocol in real blood samples needs to be further explored.

## 4. Conclusions

For the rapid detection of pathogenic bacteria in a blood sample (e.g., the blood sample of septic patients), NAAT is believed to be promising. However, the nucleic acids released from the blood cells and the PCR inhibitors existing in a whole blood sample could affect the performance of NAAT-based assays. In addition, the target DNA released by the dead bacteria in a whole blood sample could also lead to false positive detection. To address this issue, this study proposed a two-step process. First, the pre-enrichment of whole blood samples using well-known techniques (e.g., immuno-magnetic microbead-based separation) was utilized to remove the majority of unwanted blood cells and reduce the working volume. In the second step, an ODEP microfluidic system was designed to further isolate and purify the live bacteria from the remaining blood cells. In the proposed ODEP microfluidic system, a dynamic circular light image array consisting of multicolumn circular light images was designed to remove the unwanted blood cells remaining in the treated blood sample in a continuous manner. In this work, the optimum operation conditions (i.e., the 45 μm diameter of the circular light image, 10 μm gap between circular light images, the 200 μm s^−1^ moving velocity of the circular light images and the 1.0 μL min^−1^ of the sample flow rate) and the design of the dynamic circular light image array (i.e., the array with jagged front line) were determined based on a series of experimental works. The performance for the isolation and purification of bacteria was evaluated. The results revealed that the proposed ODEP microfluidic system was able to harvest the live bacteria with a purity as high as 90.5~99.2% within the experimental conditions explored. Overall, the presented method was proven to be capable of isolating and purifying high-purity live bacteria without causing damage to the co-existing cells. This technical feature is found to be valuable for the subsequent NAAT-based bacteria detection, in which the interferences caused by the nucleic acids released from the dead cells or dead bacteria could be eliminated.

## Data Availability

Not applicable.

## Supplementary Materials

The following supporting information can be downloaded at: https://www.mdpi.com/article/10.3390/bios13110952/s1, Figure S1: (a) The microscopic observation of cells in 1 μm of prepared sample (containing the SW620 cancer cells prestained with calcein red-orange-dye) before loading into the ODEP microfluidic system, (b) the photograph of bacteria culture of the processed sample (the 1:1 case study) harvested from the downstream part of main microchannel and (c) the microscopic observations (six views) of cells in the processed sample harvested from the downstream part of main microchannel (demonstrating no cell was found in the processed sample); Video S1: The video clip of the flowing cells trapped and transported by the designed dynamic circular light image array with uniform front line design; Video S2: The video clip of the flowing cells trapped and transported by the designed dynamic circular light image array with jagged front line design; Video S3: The video clip demonstrating the use of the proposed ODEP-based method for the isolation and purification of bacteria from a mixture sample containing cells and bacteria.
