# Supplementary material for: Development of an Optically Induced Dielectrophoresis (ODEP) Microfluidic System for High-Performance Isolation and Purification of Bacteria
Source: Biosensors (Basel). 2023 Oct 25;13(11):952. doi: 10.3390/bios13110952 (PMC10669672; doi:10.3390/bios13110952)
Supplement: Supplementary file 1 [file biosensors-13-00952-s001.zip › Supplementary Materials.pdf]

biosensors

MDPI

Article

Development of an optically induced dielectrophoresis (ODEP) microfluidic system for high-performance isolation and purification of bacteria

Po-Yu Chu[1,†], Chia-Ming Yang[2,3,4,5,6,†], Kai-Lin Huang[1], Ai-Yun Wu[1], Chia-Hsun Hsieh[7,8], A-Ching Chao[9,10*], Min-Hsien Wu[1,7,8]**

[1] Graduate Institute of Biomedical Engineering, Chang Gung University, Taoyuan City, 33302, Taiwan; d000018394@cgu.edu.tw; M1031009@cgu.edu.tw; M1031001@cgu.edu.tw;
[2] Department of Electronic Engineering, Chang Gung University, Taoyuan City, 33302, Taiwan; cmyang@mail.cgu.edu.tw
[3] Institute of Electro-Optical Engineering, Chang Gung University, Taoyuan City, 23652, Taiwan
[4] Biosensor Group, Biomedical Engineering Research Center, Chang Gung University, Taoyuan City, 33302, Taiwan
[5] Department of Neurosurgery, Chang Gung Memorial Hospital at Linkou, Taoyuan City, 33302, Taiwan
[6] Department of Materials Engineering, Ming Chi University of Technology, New Taipei City, 23652, Taiwan
[7] Division of Hematology/Oncology, Department of Internal Medicine, Chang Gung Memorial Hospital at Linkou, Taoyuan City, 33302, Taiwan; wisdom5000@cgmh.org.tw
[8] Division of Hematology/Oncology, Department of Internal Medicine, New Taipei Municipal Hospital, New Taipei City, 23652, Taiwan
[9] Department of Neurology, Kaohsiung Medical University Hospital, Kaohsiung City, 80756, Taiwan
[10] Department of Neurology, College of Medicine, Kaohsiung Medical University, Kaohsiung City, 80756, Taiwan
* Correspond: achch@cc.kmu.edu.tw; Tel.: +886-7-3121101 ext. 6833
** Correspondence: mhwu@mail.cgu.edu.tw; Tel.: +886-3-2118800 ext. 3599
† Chu and Yang contributed equally to this work.

Supplementary Figure

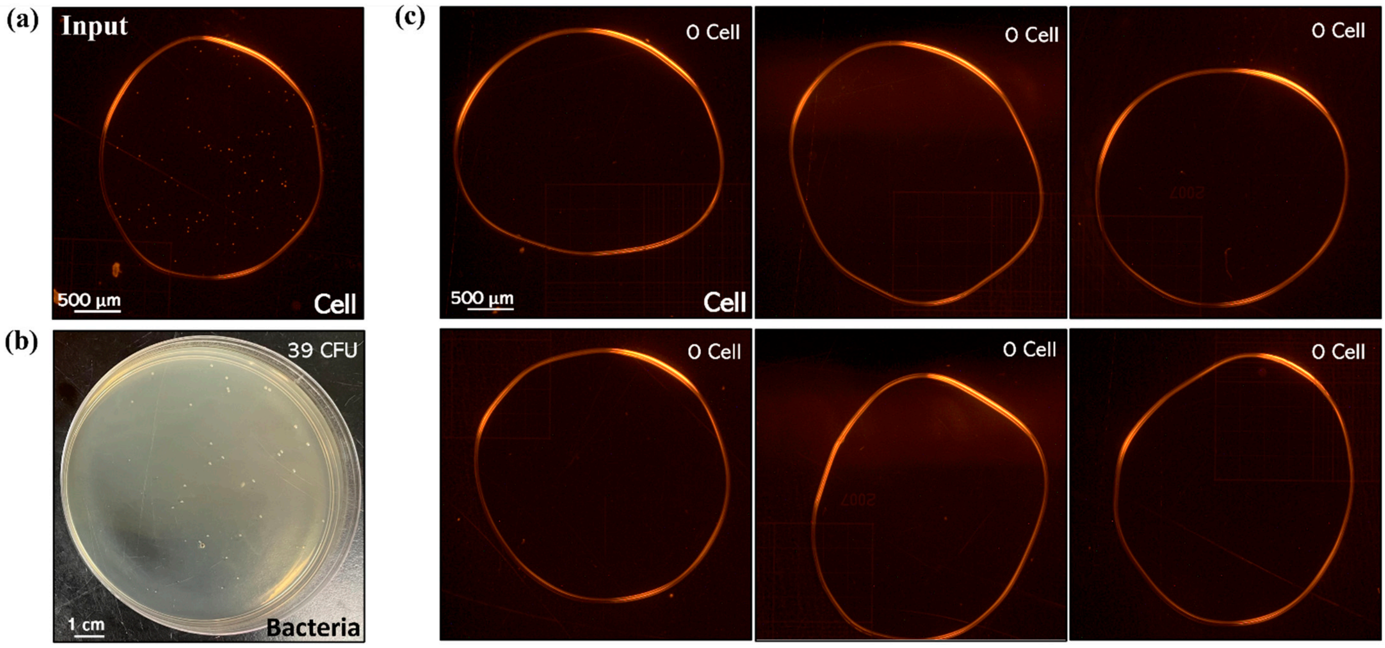

Figure S1. **(a)** The microscopic observation of cells in 1μm of prepared sample (containing the SW620 cancer cells pre-stained with calcein red-orange-dye) before loading into the ODEP microfluidic system, **(b)** the photograph of bacteria culture of the processed sample (the 1:1 case study) harvested from the downstream part of main microchannel, and **(c)** the microscopic observations (six views) of cells in the processed sample harvested from the downstream part of main microchannel (demonstrating no cell was found in the processed sample).

Supplementary Video

Supplementary video S1 The video clip of the flowing cells trapped, and transported by the designed dynamic circular light image array with uniform front line design
Supplementary video S2 The video clip of the flowing cells trapped, and transported by the designed dynamic circular light image array with jagged front line design
Supplementary video S3 The video clip demonstrating the use of the proposed ODEP-based method for the isolation and purification of bacteria from a mixture sample containing cells and bacteria
